# Ultrastructural Morphometry of Mitochondria: Comparison Between Conventional Operator‐Dependent and Artificial Intelligence (AI)‐Operated Machine Learning Methods

**DOI:** 10.1002/jemt.24866

**Published:** 2025-03-29

**Authors:** Daniele Nosi, Daniele Guasti, Alessia Tani, Sara Germano, Daniele Bani

**Affiliations:** ^1^ Imaging Platform, Dept. Experimental & Clinical Medicine University of Florence Florence Italy

**Keywords:** machine learning, mitochondria, morphometry, ultrastructure

## Abstract

Morphometric analysis of digital images is fundamental to substantiate the visual observations with objective quantitative data suitable for statistical analysis. The recent advances in artificial intelligence (AI) have allowed the development of machine learning (ML) protocols for automated morphometry. Transmission electron microscopy (TEM) morphometry requires that the ultrastructural details be recognized and interpreted by a trained observer; this makes adapting AI‐operated protocols to TEM particularly challenging. In this study, we have checked the accuracy of the results of mitochondrial morphometry yielded by a ML method by comparison with those obtained manually by a trained observer on the same TEM micrographs (magnification ×50,000) of cultured cells with different energy metabolism (overall *n* = 26). The measured parameter was the ratio between the total length of the mitochondrial cristae and the corresponding mitochondrial surface area (C/A ratio), directly related to mitochondrial function. No statistically significant correlation (Pearson's test) was found between the two methods in any of the experiments. Only in a few micrographs were the values similar (*n* = 3) or very close (*n* = 2) to be comprised within the s.e.m. of their experimental group. Moreover, as judged by the s.d. comparison, the scatter of values was more prominent with the ML‐operated than with the manual method. Conceivably, this outcome is because many ultrastructural details of the cell organelles are similar, for example, the membrane section profiles, and can only be properly recognized and distinguished by an experienced observer, while the current ML protocols still cannot.

## Graphical abstract

A comparison of the results of two methods, operator‐ versus machine learning (ML)‐made, for transmission electron microscopic (TEM) morphometry of mitochondria showed no statistical correlation between the two methods due to insufficient specificity of the latter.

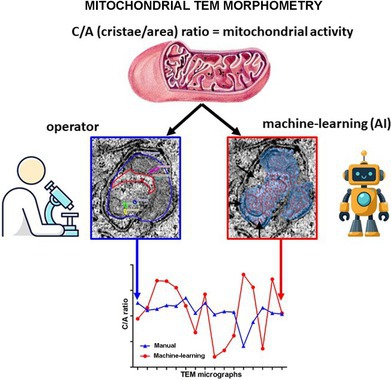

**Summary**

- We compared two methods for ultrastructural morphometry of mitochondria (from cells cultured in different metabolic conditions), conventional operator‐dependent and artificial intelligence (AI)‐based machine learning (ML), to evaluate the latter's precision and reliability.
- The ratio between the mitochondria's length of cristae and whole surface area (C/A), which is related to mitochondrial function, was calculated using either method.
- Comparison of the results showed no statistical correlation between the operator‐dependent and ML methods due to insufficient specificity of the latter.
- Analysis of these results yields helpful information to develop reliable ML protocols for ultrastructural morphometry of mitochondria.

## 1 Introduction

The recent burst in developing artificial intelligence (AI) resources with ever‐increasing performances has critical reflections on biomedical sciences. In particular, machine learning (ML) has been successfully tested to analyze digital images, such as brain magnetic resonance imaging for neuropsychiatric disorders or functional radiologic imaging of cardiovascular diseases, as a diagnostic support with three clinical goals: disease risk assessment, subtyping, and treatment decision (Aydin et al. 2019; Gopalan and Gibbs 2020). In a similar perspective, ML protocols have been applied to analyze digital microphotographs, generated by either conventional light and fluorescence microscopy or confocal laser scanning microscopy, in support of histopathology for clinical or veterinary purposes, for instance, to detect nuclear shape abnormalities as an index of tissue malignancy (Haghofer et al. 2025; dos Santos et al. 2024), or to measure nerve fiber dimensions in biopsies taken from patients with peripheral neuropathies (Ono et al. 2024). A similar approach has been used for research purposes, for example, to analyze axon regeneration/remyelination in cross‐sections of injured nerves in rats (Daeschler et al. 2022) or to investigate the relationships between reactive glial cells and amyloid plaques in 3D confocal images of the hippocampus in a mouse model of Alzheimer's disease (Lana et al. 2023).

Morphometric analysis of digital images is fundamental to substantiate the visual observations with objective quantitative data suitable for statistical analysis. Many morphometrical software tools are available, some of which, such as ImageJ/FIJI (http://imagej.org, NIH, USA), are open‐source and continuously improved by user‐developed add‐ons. Although these tools are a tremendous help, allowing accurate, reproducible, and reliable measures to be obtained, they require to be manually operated, which usually implies a long working time for a complete analysis, especially for multiple samples or large images with many measurable details. This is particularly true for the digital black‐and‐white micrographs of transmission electron microscopy (TEM), in which the ultrastructural details need to be recognized and interpreted by a trained observer before being selected adequately for morphometric analysis. The ultrastructural features of the cell mitochondria are a paradigm of TEM morphometry. It is well known that the inner mitochondrial membrane, where the cytochrome chain and ATP‐synthase are located, is a dynamic structure that can vary its surface with mitochondrial activity, resulting in changes in the overall extension of its infoldings, the so‐called mitochondrial cristae. Thus, the relative density of the mitochondrial cristae can be a reliable parameter of the functional activity of these organelles, which can vary depending on the different states of the cells, for example, metabolic activation, differentiation, quiescence, senescence, etc. This parameter can be quantitated by the ratio between the total length of the mitochondrial cristae and the corresponding mitochondrial surface area in longitudinal, oblique, or cross sections of mitochondria, measured by an image analysis software on TEM micrographs of the cells under study (Maneschi et al. 2016; Buonvicino et al. 2024). This procedure is particularly time‐consuming and requires a dedicated trained operator.

No ML protocols have yet been developed for ultrastructural morphometry. Therefore, in the present study, we aim to compare the mitochondrial morphometry results obtained by the canonical operator‐dependent manual method and by AI software (AIVIA, Leica Microsystems, Milan, Italy), initially developed for the analysis of confocal microscopy images, instructed to self‐recognize and analyze the noted mitochondrial parameters.

## 2 Materials and Methods

### 2.1 Cell Culture and Preparation

Human prostate cancer cells (PC3 line, ATCC) were grown in RPMI plus 10% FBS, 2 mmol/L l‐glutamine, and 1% penicillin/streptomycin at 37 °C and 5% CO_2_. A first set was cultured in attached conditions in plain 6‐well plates with regular metabolic activity (2D), and a second set was grown in detached conditions in soft agar‐coated 6‐well plates to induce enhanced metabolic activity (3D). The cells were detached from the culture plates with 0.25% trypsin/EDTA solution, pelleted by centrifugation at 600 g, fixed in Karnowsky's fluid, postfixed in OsO_4_, and embedded in epoxy resin. Ultrathin sections were cut and observed at a JEM‐1010 TEM (Jeol, Tokyo, Japan) at 80 kV.

### 2.2 Manual Morphometry

This was carried out on TEM micrographs, magnification ×50,000, of randomly chosen cytoplasmic areas showing at least 1 mitochondrial section profile. The micrographs were taken with a high‐resolution CCD camera (Veleta, EMSIS GmbH, Münster, Germany) interfaced with the TEM (image dimensions: 2048 × 2048 pixels; 1.38 pixels per nanometer). Ten and 16 micrographs were taken from the 2D and 3D experimental groups, respectively. On each micrograph, the mitochondrial sections were analyzed by a proprietary image analysis software (iTEM, EMSIS GmbH) by an expert operator (D.G.), who measured the cristae's overall length (nm) and whole surface area (nm^2^) of each mitochondrial profile. Then, the cristae/area ratio (C/A) was calculated.

### 2.3 Machine Learning Morphometry

This was carried out on the same TEM micrographs used for manual morphometry. The mitochondrial sections were analyzed on each micrograph using the AIVIA software (Leica Microsystems). The first step exploited the Cellpose Enhancement tool, which utilizes pretrained deep‐learning models, to compensate for two main issues: (i) the analyzed images exhibited uneven contrast, and (ii) the mitochondria showed varying electron density of the inner matrix. These issues limited the direct application of ML models for mitochondrial membrane recognition, as these membranes were indistinguishable from those of other cell organelles, such as RER, SER, and lysosomes. The Cellpose Enhancement tool allowed the isolation of objects within the images based on their size and sphericity. A mean diameter ranging from 151.72 to 206.90 nm (corresponding to 110–150 pixels) was set to select mitochondria. The selection of objects was further refined by adjusting the percentile of the probability map, which varied between the 92nd and 97th percentiles, depending on the image quality and mitochondrial density. The identified objects were enclosed in regions of interest (ROIs), and for each of them, the surface area was calculated.

In the second step, mitochondrial cristae were segmented within the previously defined ROIs using ML models specifically trained for this purpose. Segmentation enabled the measurement of the cristae's length. Finally, the cristae/area ratio (C/A) was calculated.

### 2.4 Statistic Analysis

This was performed with Prism 5.0 statistical software (GraphPad Software, Boston, MA, USA). A Pearson's correlation test was performed for either the 2D or 3D experiments to compare the paired data obtained from each micrograph by manual and ML‐made analyses and to assess whether the two methods yielded comparable results. Moreover, for each experimental group, the mean ± s.e.m. was calculated; the statistical comparison between the means obtained by manual and ML‐made analyses was carried out by Student's *t*‐test for paired values, separately for the 2D and 3D experiments. A *p*‐value ≤ 0.05 was assumed as significant.

## 3 Results

In the cross sections of mitochondria, recognizable as such in the ×50,000 ultrastructural micrographs, the manual and ML‐operated methods generated similar raw data, consisting of linear length measures of the mitochondrial cristae and surface area measures of the sectioned mitochondria. For each mitochondrial section, the ratio between the overall length of cristae and the whole surface area (C/A) was calculated and assumed to be a test parameter directly proportional to mitochondrial functional activity.

**FIGURE 1:**
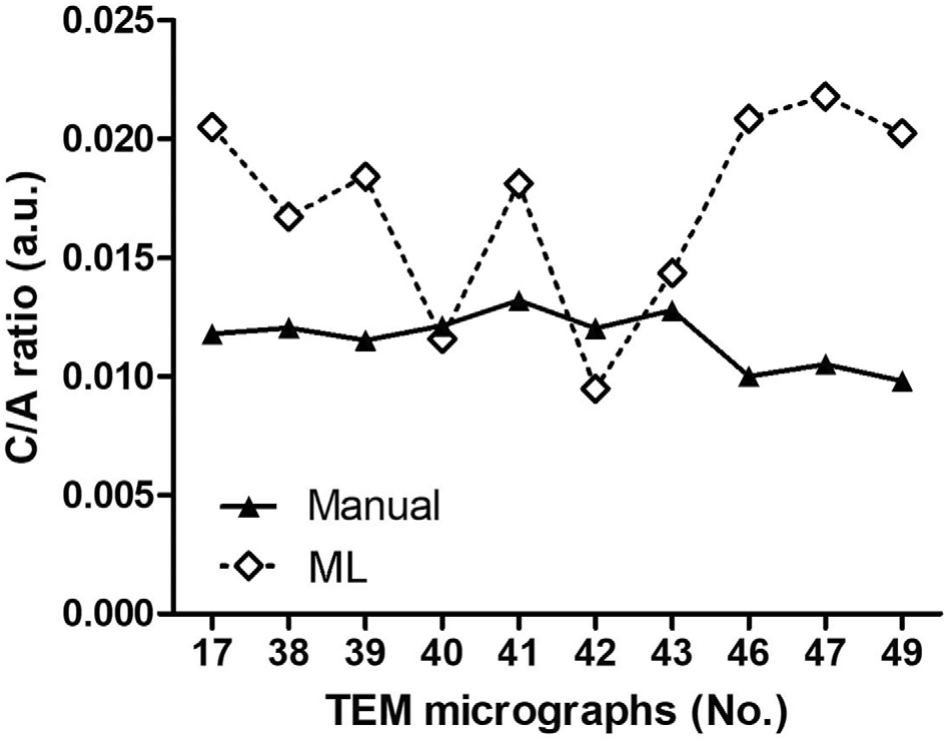
Plot of the cumulative C/A ratios of the mitochondria from the 10 micrographs of the 2D experiment, calculated by the ML‐operated and manual method (a.u.: Arbitrary units).

**FIGURE 2:**
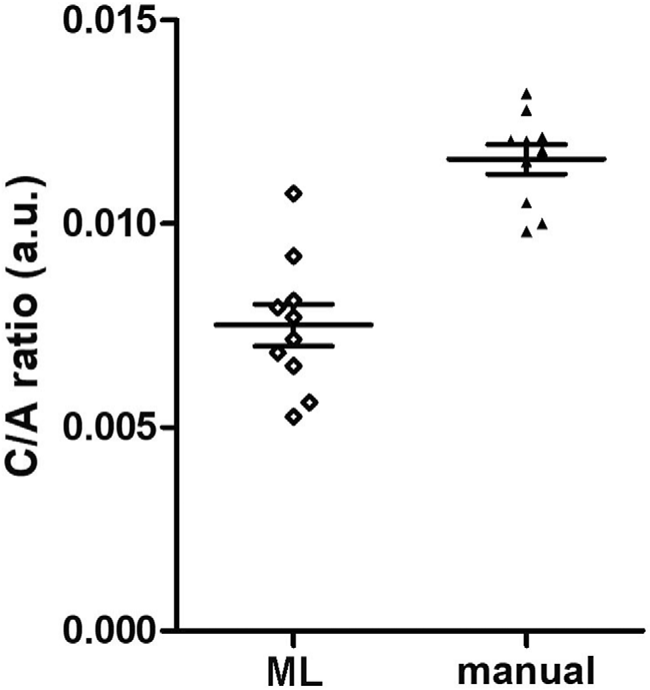
Scattergram of the values of the mitochondrial C/A ratios evaluated by the ML‐operated and manual method in the 10 micrographs of the 2D experiment. Horizontal lines represent the mean ± s.e.m. (a.u.: Arbitrary units).

A comparison of the C/A ratios in the 10 micrographs from the 2D experiments yielded by manual and ML‐operated methods (Figure 1) showed no statistically significant correlation (Pearson's test: *r* = −0.57, *p* = 0.09). In particular, only in 1 micrograph (No. 40) were the values similar (0.0116 vs. 0.0121), and only in another (No. 43) were the values close enough (0.0144 vs. 0.0128) to be comprised within the s.e.m. (± 0.0013) of the experimental group. Moreover, the scatter of the values from the 10 micrographs was markedly larger with the ML‐operated than with the manual method, as indicated by comparison of the s.d. of the means (ML: 0.0172 ± 0.0042; manual: 0.0120 ± 0.0011) (Figure 2).

**FIGURE 3:**
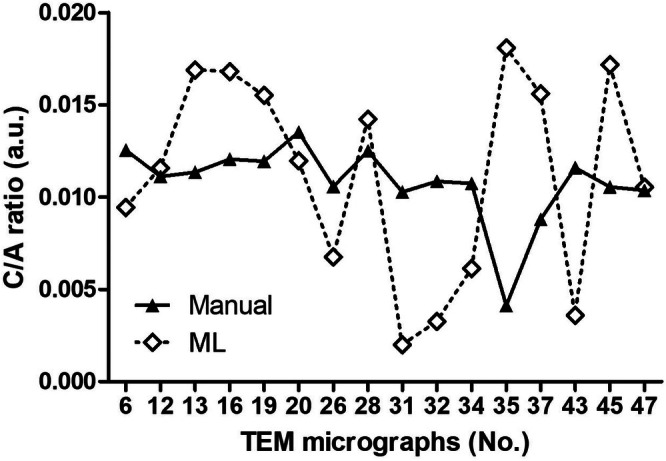
Plot of the cumulative C/A ratios of the mitochondria from the 10 micrographs of the 2D experiment, calculated by the ML‐operated and manual method (a.u.: Arbitrary units).

**FIGURE 4:**
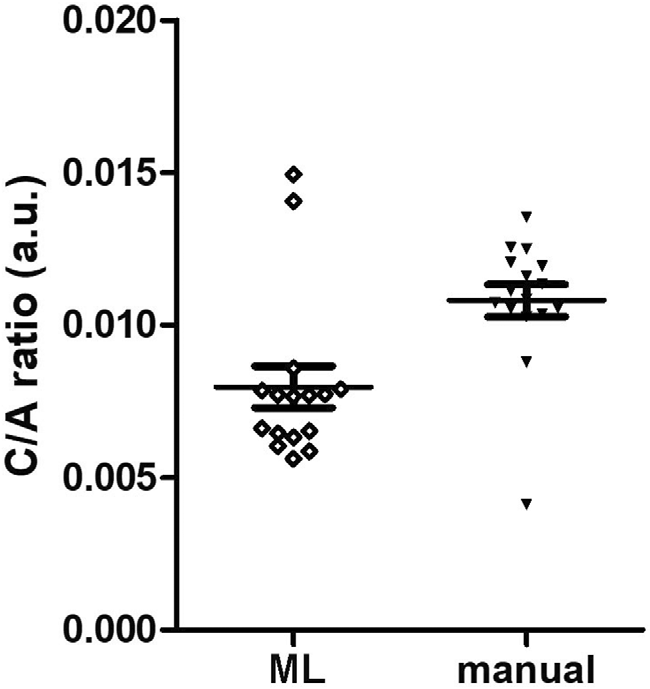
Scattergram of the values of the mitochondrial C/A ratios evaluated by the ML‐operated and manual method in the 16 micrographs of the 3D experiment. Horizontal lines represent the mean ± s.e.m. (a.u.: Arbitrary units).

A comparison of the C/A ratios in the 16 micrographs from the 3D experiments yielded by manual and ML‐operated methods (Figure 3) also failed to reveal any statistically significant correlation (Pearson's test: *r* = −0.21, *p* = 0.43). In particular, only in 2 micrographs (No. 12, 47) were the values similar (0.0116 vs. 0.0111 and 0.0105 vs. 0.0104) and only in another (No. 28) were the values close enough (0.0142 vs. 0.0125) to be comprised within the s.e.m. (±0.0014) of the experimental group. Moreover, the scatter of the values from the 16 micrographs was more prominent with the ML‐operated than with the manual method, as indicated by comparison of the s.d. of the means (ML: 0.0112 ± 0.0014; manual: 0.0108 ± 0.0021) (Figure 4).

**FIGURE 5:**
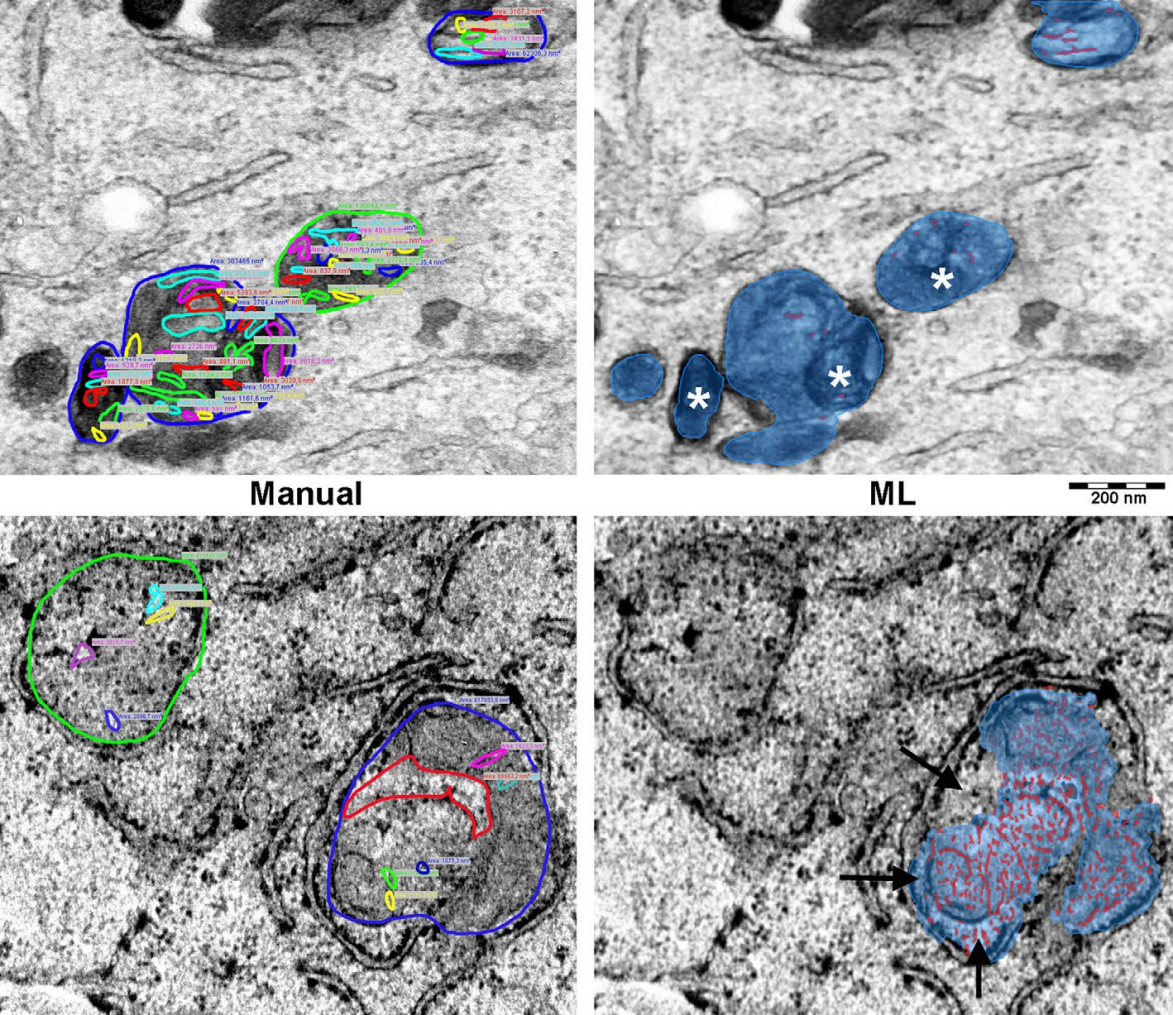
Visual comparison of the same TEM micrographs showing different section profiles of mitochondria analyzed by manual or ML‐operated morphometry. In these latter images, the mitochondrial area is in blue, while the cristae are in red. The upper panels show an example of an underestimation of the cristae's overall length by ML morphometry, while the lower panels show an overestimation. The asterisks indicate some areas with undetected mitochondrial cristae; the arrows point at details erroneously included or excluded from the mitochondrial surface area. Magnification ×50,000.

Figure 5 shows a visual comparison of the graphical output yielded by the manual and ML‐operated methods on the same micrographs, selected among those that showed the most remarkable differences, in plus or minus, in C/A ratios between the two methods. There were slight differences in the surface areas of some mitochondrial sections between the two methods. Conversely, the ML‐operated morphometry yielded underestimated measures of the cristae's overall length in the low‐contrast figures with faint electron density differences between the cristae's membranes and the mitochondrial matrix, whereas it yielded overestimated measures in the high‐contrast figures, where the granular appearance of the matrix was erroneously assumed as membrane profiles.

## 4 Conclusions

TEM is experiencing a revival of interest in bio‐medicine, particularly in the last two decades, along with the development of new research fields in nanoscience and nanotechnology (Malatesta 2016). As for other digital imaging techniques, the possibility of supporting visual observation with objective quantitation by morphometrical methods can be a valuable addition exploitable for both research and diagnostic purposes. In this context, the ultrastructural analysis of mitochondria can support functional studies on the cell's metabolic activity since the relative extension of the mitochondrial cristae can be related to the degree of oxidative phosphorylation (et al., 2023). This parameter can be quantitated morphometrically by the C/A ratio, which has been used as a reliable marker for cell activation (Maneschi et al. 2016; Buonvicino et al. 2024) and differentiation (Comeglio et al. 2018). Numerous mitochondria must be analyzed for each experimental group to collect enough measurements for statistical purposes and compensate for the differences dependent on the section profiles. For this reason, the set‐up of AI‐operated ML protocols would be desirable to expedite the morphometrical procedure. Still, their reliability needs to be checked by comparison with the manual method performed by a trained observer. The current findings show no sufficient, statistically significant correlation between the manual and the ML‐operated methods. With the settings used to instruct the AIVIA AI software, a broad scatter of the measured values exists between the micrographs from the same experimental group, both 2D and 3D. A careful comparison between the micrographs showing the most prominent divergences between the two methods has revealed that the AI cannot discriminate and measure the oblique or tangential cristae profiles, at variance with the experienced observer who did the same analysis manually. This limitation was in part overridden by instructing the software only to include the standard mitochondrial section profiles, that is, longitudinal or slightly oblique. This choice, however, reduced the sampling size with obvious repercussions on the measure's accuracy and statistical power. Another issue depends on differences in the contrast of the TEM micrographs, which, in our analysis, hampered the AI's ability to correctly discriminate between the cristae's membrane and the inner mitochondrial matrix based on their different electron density.

In conclusion, this study indicates that the setting of AI software to perform ML‐operated morphometry on TEM micrographs is particularly challenging since: (i) the black‐and‐white ultrastructural images do not allow a precise selection of the cell details based on their colors, as can be done with conventional light microscopic or confocal images; (ii) many details of the cell organelles are similar, for example, the membrane section profiles, and require to be precisely recognized and distinguished by an experienced observer. Nonetheless, the continuing, swift progress in AI development should be capable of overriding these limitations in the future. It should also be pointed out that an ML‐operated method is substantially faster than a manual one, allowing for a far greater number of TEM micrographs to be analyzed in a shorter time. This would result in a broader sample size, easily overriding the negative impact of accuracy bias and the related scattering of values on statistical analyses. Nonetheless, the present study highlights the need for a careful check of the data generated by an ML‐based image analysis procedure before assuming it to be scientifically valid. Such a check is crucial to identifying and correcting biases in its algorithm to improve its accuracy and reliability.

The present results also add a tile to the complex mosaic concerning the use and misuse of AI, which is becoming a significant issue in the general digital security framework (Radanliev 2024). AI software for image analysis is being developed for many purposes to accelerate or improve the capacities of specifically trained operators, for example, in support of clinical radiological or histopathological diagnosis (Aydin et al. 2019; dos Santos et al. 2024). This raises the need to find an equilibrium between the fore promoted by AI software companies aiming to lead (and earn the proper profit from) the introduction of such technologies in healthcare practice and the aft suggested by independent research aiming to verify their reliability and, hence, safety. Public health authorities should be aware and increasingly involved in finding and regulating such equilibrium.

## Author Contributions

**Daniele Nosi:** conceptualization, investigation, methodology, data curation, formal analysis, software, writing – review and editing. **Daniele Guasti:** investigation, methodology, validation, formal analysis, data curation, software. **Alessia Tani:** conceptualization, investigation, methodology, writing – review and editing, formal analysis, data curation. **Sara Germano:** investigation, methodology, validation. **Daniele Bani:** conceptualization, funding acquisition, writing – original draft, methodology, validation, formal analysis, data curation, supervision, investigation.

## Conflicts of Interest

The authors declare no conflicts of interest.

## Data Availability

The data that support the findings of this study are available from the corresponding author upon reasonable request.
